# Quality of Distance Learning After One and a Half Year From Its Integration Due to the COVID-19 Pandemic: A Cross-Sectional Study at the University of Jordan

**DOI:** 10.7759/cureus.32642

**Published:** 2022-12-17

**Authors:** Ahmad A Toubasi, Sarah M Al-Harasis, Yazan Y Obaid, Farah H Albustanji, Heba M Kalbouneh

**Affiliations:** 1 Department of Medicine, Faculty of Medicine, University of Jordan, Amman, JOR; 2 Department of Anatomy, Faculty of Medicine, University of Jordan, Amman, JOR

**Keywords:** deles tool, university students, distance learning, education, covid-19

## Abstract

Background

The coronavirus disease 2019 (COVID-19) pandemic mandated the change from face-to-face learning to distance learning. As a result, the quality of distance learning worldwide is questionable.

Objectives and aims

The aim of this study is to investigate the quality of distance learning among university students at the University of Jordan, as well as its determinants and obstacles.

Methods

A questionnaire-based cross-sectional study was conducted among undergraduate students at the University of Jordan from May 29 to July 11, 2021. Using a quantitative approach, students’ attitudes toward and perceptions of online and distance education were analyzed. The Distance Education Learning Environments Survey (DELES) tool was used to evaluate the quality of distance learning during COVID-19 pandemic. IBM SPSS Version 25 was used for data analysis.

Results

The total number of participants was 486, and the mean total DELES score was 61.6 ± 24.6. The lowest mean of DELES score components was for the active learning component (6.9 ± 2.9). Moreover, 65.9% of the students were very dissatisfied or dissatisfied with the quality of distance learning. The quality of distance learning derived by the total DELES score was determined by several general demographics, and students’ perceptions and attitudes.

Conclusions

The present study showed a low quality of distance learning and high levels of dissatisfaction among students at the University of Jordan. Thus, improvement of distance learning quality by filling its infrastructural defects and the implementation of its adjunct tools are required. In addition, we recommend collaboration with regional and international educational institutions to improve the quality of distance learning.

## Introduction

The coronavirus disease 2019 (COVID-19) is a highly contagious disease, which was first identified in December 2019. Since then, it had spread worldwide and was officially declared a pandemic by the World Health Organization (WHO) [1]. COVID-19 posed an enormous burden on almost all of Jordan’s different departments including the economy, general health, and education system. Therefore, in order to limit further spread of the disease and minimize the impact on education, schools and universities had resorted to distance learning from March 2020 until the beginning of 2022.

Distance learning was defined as the provision of educational programs through electronic systems [2]. There were several advantages to distance learning, such as lower costs, widespread distribution, increased accessibility to information, frequent content updates, and personalized instruction in terms of content and pace of learning [3]. Furthermore, the interactivity and ability to link educational programs to past experiences and specific requirement could be achieved through distance learning [4]. On the other hand, there were some barriers to distance learning, including technology‐related costs, technical problems, decreased direct interaction, and lack of relations with other learners. Additionally, it was previously reported that incorporating social media in distance learning was only beneficial for theoretical courses but not feasible for practical courses [5]. Lastly, students might suffer from low motivation to learn and a decrease in the feeling of competitiveness with other learners during distance learning [6-8].

Since the start of the pandemic, several studies had investigated the quality of distance learning. In one cross-sectional study, results showed that only 26.8% of the students were satisfied with distance learning [9]. Furthermore, the inclination toward distance learning was associated with several factors such as the year of study, as students in first years of study showed higher desire toward distance learning compared to their counterparts [10]. The most reported benefits in the literature were time saving and flexibility of class time, whereas poor internet coverage, limitation in internet data packages, and variation in educational platforms were the most reported hurdles [9]. Another study showed that 40.1% of students had some difficulty understanding classes, while 24.6% faced an even greater difficulty. This was due to hardships in maintaining a study routine, learning in the absence of monitoring from faculty members, and learning consolidation [11].

Although numerous studies investigated the quality of distance learning amid the COVID-19 pandemic, all of them were conducted at the beginning of the distance learning experience. Consequently, we decided to conduct a study that aims to (1) assess the quality of distance learning after a year and a half from its start, (2) establish determinants of distance learning quality, and (3) recognize obstacles and difficulties of distance learning.

## Materials and methods

Study design and setting

We conducted a cross-sectional study at the University of Jordan from May 29 to July 11, 2021. The University of Jordan is a public university located in Amman, Jordan. It offers more than 250 programs from 24 schools in various disciplines. Moreover, the student body within the university is composed of diverse ethnic and socioeconomic backgrounds. The University of Jordan implements the grade point average (GPA) scale as a part of its educational system. The cumulative GPA is out of 4 and described as follows: 4.00-3.65, excellent; 3.64-3.00, very good; 2.99-2.5, good; 2.49-2.00, pass; and less than 2.00, fail).

Sample size determination and sampling procedure

We determined the minimum sample size using the Raosoft online sample calculator. The calculation of sample size was based on a response rate of 50%, a margin of error of 5%, and a confidence interval of 95%. In the academic year 2020/2021, the number of students enrolled at the University of Jordan was 40,142 in total, and the calculated sample size was 381. Therefore, using convenient sample techniques, we were able to collect 556 responses. Seventy responses were excluded due to absence of consent and incomplete filling of one or more of Distance Education Learning Environments Survey (DELES) questions.

Study subjects and eligibility criteria

All the university students were eligible for participation in this study except higher degree students and medical field clinical students because they experienced some form of face-to-face education.

Outcome measures

An online, self-administered questionnaire was created using Google forms and shared on various groups for each faculty on social media platforms. The questionnaire was designed in English, translated to Arabic, and then back-translated by another author to English in order to ensure the retained meaning of the original questionnaire. The questionnaire was composed of 58 questions divided into four sections. The first section was on the demographics of the students. The second section consisted of questions regarding the students’ attitudes during the distance learning period. The third section evaluated the students’ perception of distance learning. Finally, DELES was used in the last section to assess the quality of virtual learning environments. The DELES is a valid and reliable tool used for the evaluation of the quality of distance learning. This tool is composed of 34 questions divided into six components that assess for instructor support, student interaction and collaboration, personal relevance, authentic learning, active learning, and student autonomy. Each item of this tool is scored using a five-point Likert scale that ranges from never to always. The alpha reliability coefficient for each of its components ranged between 0.75 and 0.94 [12]. The questionnaire was presented on Google forms and shared with students via social media platforms.

Data analysis

The participants’ data were entered into a spreadsheet and analyzed using the SPSS Version 25 (IBM Corp., Armonk, NY, USA). Categorical variables were expressed as counts and percentages, whereas continuous variables were expressed as mean ± SD, median, minimum, and maximum. To identify the predictors of DELES scores, T-test and one-way analysis of variance (ANOVA) test were used for categorical predictors with two or three groups, respectively. Tukey’s HSD (honestly significant difference) test was used for post-hoc comparison between the different groups. Pearson’s correlation coefficient was used to explore the relationship between continuous variables and DELES scores. Across all tests, the predictors were considered significantly associated with DELES score when the p-values were less than 0.05.

Ethical approval

The Institutional Review Board (IRB) at our institution reviewed and approved the conductance of this study. The questionnaire opened with a brief introduction about the aims of the study, and a consent statement was presented and confirmed by the participants. Confidentiality was maintained at all times.

## Results

Demographic characteristics

The total number of participants in this study was 486. Of them, 67.3% were females (327/486), and 32.4% of the study participants were in their first year of study (157/486). The most represented faculty in this study was medicine (31.5%), and most of the participants had a GPA higher than 3.00 (79.4%). The mean age was 20.2 ±1.6 years. The mean number of courses taken during distance learning was 14.1± 6.6, while the maximum number of courses that were taken during distance learning was 30 (Table 1).

**Table 1:** Demographic characteristics of participants GPA, grade point average

Variable	Response	Frequency (n = 587)	Percentage (%)
Sex	Male	159	32.7
	Female	327	67.3
Year of study	First year	157	32.4
	Second year	99	20.5
	Third year	115	23.8
	Fourth year	81	16.7
	Fifth year	24	5.0
	Sixth year	8	1.7
GPA	Less than 2.00	4	0.8
	2.00-2.49	19	4.0
	2.50-2.99	76	15.8
	3.00-3.64	221	45.9
	3.65-4.00	161	33.5
Faculty	Faculty of Medicine	154	31.8
	Faculty of Nursing	17	3.5
	Faculty of Pharmacy	36	7.4
	Faculty of Dentistry	35	7.2
	Faculty of Rehabilitation Sciences	16	3.3
	Faculty of Arts and Designing	3	0.6
	Faculty of Science	23	4.7
	Faculty of Agriculture	16	3.3
	Faculty of Engineering	59	12.2
	Faculty of Information Technology	23	4.7
	Faculty of Business	16	3.3
	Faculty of Law	20	4.1
	Faculty of Educational Sciences	10	2.1
	Faculty of Physical Education	9	1.9
	Faculty of Islamic Studies	11	2.3
	Faculty of International Studies	2	0.4
	Faculty of Foreign Languages	28	5.8
	Faculty of Archeology and Tourism	4	0.8
	Faculty of Arts	3	0.6
Variable	Mean	SD	Range
Age (years)	20.2	1.6	14.0-35
Courses	14.1	6.6	1-30

Students’ attitudes during the distance learning period

The participants who spent more than 4 hours studying and 3-4 hours attending lectures accounted for 37.3% and 42.4%, respectively. More than 65% of the participants stated that they were dissatisfied or very dissatisfied with distance learning. Moreover, 80.3% of the participants expressed that they were bored of distance learning to a degree that stopped them from attending lectures. The percentage of participants who self-perceived themselves as burnt out was 86.5%, and almost all of them (85.6%) considered distance learning as the cause. In addition, 63% reported that they frequently attended their lectures while not paying attention, and 13% of the students who have driving licenses responded that they frequently attended lectures while driving. Also, 61% of the participants thought to have social anxiety, and 59.7% thought that the source of their social anxiety was distance learning. In the students’ self-assessment comparison of their previous studying level (before distance learning) and their current studying level (with distance learning), 60.4% found their level to be worse than before, and 40.7% of the participants found that their current grades level was worse than before distance learning (Table 2).

**Table 2:** Students’ attitudes during the distance learning period due to the COVID-19 pandemic

Variable	Response	Frequency (n = 587)	Percentage (%)
Studying hours	Less than 1 hour	41	8.5
	1-2 hours	111	23.0
	3-4 hours	151	31.3
	More than 4 hours	180	37.3
Hours spent in attending lectures	Less than 1 hour	48	9.9
	1-2 hours	80	16.5
	3-4 hours	205	42.4
	More than 4 hours	151	31.2
Level of satisfaction with distance learning	Very dissatisfied	144	29.6
	Dissatisfied	177	36.3
	Neutral	90	18.5
	Satisfied	62	12.7
	Very satisfied	14	2.9
Bored of distance learning to a degree stopped attending lectures	Yes	391	80.3
	No	93	19.1
Burnout perceiving	Yes	418	86.5
	No	65	13.5
Distance learning is the cause of their burnout	Yes	417	85.6
	No	70	14.4
Thinking of quitting courses	Never	186	38.2
	Rarely	84	17.2
	Sometimes	123	25.3
	Often	53	10.9
	Always	41	8.4
Irregularity in lecture times	Never	136	27.9
	Sometimes	243	49.9
	Frequently	108	22.2
Attended the lecture while not paying attention to it	Never	26	5.3
	Sometimes	154	31.7
	Frequently	306	63.0
Attended lecture while driving	Never	287	62.0
	Sometimes	116	25.1
	Frequently	60	13.0
Comparison between their level of studying during distance learning with their previous level	Worse than before	233	60.4
	Same as before	99	25.6
	Better	54	14.0
Comparison between their academic grades during distance learning with their previous grades	Worse than before	160	40.7
	Same as before	166	42.2
	Better	67	17.0
How much do you agree with this sentence: “in distance learning I barely communicate with my colleagues”	Strongly disagree	20	4.1
	Disagree	61	12.5
	Neutral	101	20.7
	Agree	153	31.4
	Strongly agree	152	31.2
Social anxiety perceiving	Yes	297	61.0
	No	187	38.4
Distance learning is the cause of social anxiety	Yes	285	59.7
	No	192	40.3
Smartphone use	Less than 1 hour	7	1.4
	1-2 hours	26	5.4
	3-4 hours	107	22.2
	5-6 hours	124	25.7
	More than 6 hours	219	45.3

Students’ perceptions of distance learning

The vast majority (83.8%) of participants agreed that distance learning is boring. On the other hand, 59.5% of the responders denied that distance learning is time saving, and 74.7% disagreed that distance learning is flexible timewise. Only 5.3% and 6.8% of the responders thought that distance learning had better instruction and better communication with instructors, respectively. Additionally, 4.7% had better communication with their classmates. The most reported challenges by the participants of this study were poor internet coverage and limited data availability (Table 3).

**Table 3:** Students’ perceptions of distance learning

Variable	Response	Frequency (n = 587)	Percentage (%)
Studying hours	Less than 1 hour	41	8.5
	1-2 hours	111	23.0
	3-4 hours	151	31.3
	More than 4 hours	180	37.3
Hours spent in attending lectures	Less than 1 hour	48	9.9
	1-2 hours	80	16.5
	3-4 hours	205	42.4
	More than 4 hours	151	31.2
Level of satisfaction with distance learning	Very dissatisfied	144	29.6
	Dissatisfied	177	36.3
	Neutral	90	18.5
	Satisfied	62	12.7
	Very satisfied	14	2.9
Bored of distance learning to a degree stopped attending lectures	Yes	391	80.3
	No	93	19.1
Burnout perceiving	Yes	418	86.5
	No	65	13.5
Distance learning is the cause of their burnout	Yes	417	85.6
	No	70	14.4
Thinking of quitting courses	Never	186	38.2
	Rarely	84	17.2
	Sometimes	123	25.3
	Often	53	10.9
	Always	41	8.4
Irregularity in lecture times	Never	136	27.9
	Sometimes	243	49.9
	Frequently	108	22.2
Attended the lecture while not paying attention to it	Never	26	5.3
	Sometimes	154	31.7
	Frequently	306	63.0
Attended lecture while driving	Never	287	62.0
	Sometimes	116	25.1
	Frequently	60	13.0
Comparison between their level of studying during distance learning with their previous level	Worse than before	233	60.4
	Same as before	99	25.6
	Better	54	14.0
Comparison between their academic grades during distance learning with their previous grades	Worse than before	160	40.7
	Same as before	166	42.2
	Better	67	17.0
How much do you agree with this sentence: “in distance learning I barely communicate with my colleagues”	Strongly disagree	20	4.1
	Disagree	61	12.5
	Neutral	101	20.7
	Agree	153	31.4
	Strongly agree	152	31.2
Social anxiety perceiving	Yes	297	61.0
	No	187	38.4
Distance learning is the cause of social anxiety	Yes	285	59.7
	No	192	40.3
Smartphone use	Less than 1 hour	7	1.4
	1-2 hours	26	5.4
	3-4 hours	107	22.2
	5-6 hours	124	25.7
	More than 6 hours	219	45.3

DELES score and its components

The mean total DELES score was 61.6 ± 24.6. The highest mean of DELES score components was the instructor support component (14.0 ± 6.8), and the lowest mean was the active learning component (6.9 ± 2.9) (Table 4).

**Table 4:** Analysis of DELES and its component scores DELES, Distance Education Learning Environments Survey

Variable	Response	Frequency (n = 578)	Percentage (%)
Distance learning is boring	Yes	408	83.8
	No	79	16.2
Distance learning is time saving	Yes	197	40.5
	No	290	59.5
Distance learning is flexible	Yes	123	25.3
	No	364	74.7
Distance learning has better instruction	Yes	351	5.3
	No	461	94.7
Better communication with instructors	Yes	33	6.8
	No	454	93.2
Better communication with classmates	Yes	23	4.7
	No	463	95.3
Poor internet	Yes	229	47.0
	No	258	53.0
Limited internet data availability	Yes	172	35.3
	No	315	64.7
Lacking devices to use in distance learning	Yes	157	32.2
	No	330	67.8
Variety of platforms used in distance learning	Yes	109	22.4
	No	378	77.6

Determinants of the total DELES score

The results of an independent t-test showed that students who perceived themselves as burned out, socially anxious, and stopped attending their lectures had significantly lower means of total DELES scores compared to their counterparts (p<0.05). Moreover, students who experienced poor internet coverage, limited data availability, and lack of suitable devices had significantly lower means of total DELES scores compared to their counterparts (p<0.05). Also, students who reported that distance learning is boring, has worse interaction with instructors and colleagues, is not time saving, and is not timely flexible had significantly lower means of total DELES scores compared to their counterparts (p<0.05). Furthermore, one-way ANOVA test showed significant differences in the total DELES score between groups in the following variables: year of study, irregular lecture time, students’ distance learning satisfaction, thoughts about quitting courses, lacking communication with colleagues, level of study, and grades comparison before and during distance learning (p<0.05) (Table 5). The subsequent Tukey post-hoc analysis showed that the highest significant mean difference in the distance learning satisfaction variable groups was between very dissatisfied (I) and very satisfied (J) (p=0.00, mean difference (I-J)=-53.15). Similarly, the highest significant mean difference in the quitting courses variable was between never (I) and always (J) (p=0.00, mean difference (I-J)=15.03). In addition, the widest significant mean difference was between never (I) and frequently (J) in the lecture time irregularity variable (mean difference (I-J)=13.36), attending lecture while not paying attention to it variable (mean difference (I-J)=25.14), and driving while attending a lecture variable (mean difference (I-J)=21.42). The variables of the comparison of study levels (p=0.00, mean difference (I-J)=-19.77) and grading levels (p=0.00, mean difference (I-J)=-13.19) had the largest significant mean difference between worse (I) and better (J). Moreover, the largest significant mean difference was between students who strongly disagreed (I) and strongly agreed (J) in the variable about how much they agree with the statement that they barely communicate with their colleagues (p=0.00, mean difference (I-J)=31.14). (Table 6). The Pearson correlation results revealed that age and total DELES scores had a significant positive weak correlation (p=0.00, r=0.16), while number of courses was not significantly correlated to total DELES score (p>0.05, r=-0.02) (Table 7).

**Table 5:** Factors associated with the total DELES score *P-value<0.05 DELES, Distance Education Learning Environments Survey

Variable	P-value
Gender	0.21
Burnout perceiving	0.00*
Burnt out due to distance learning	0.00*
Burned out until stopped attending lectures	0.00*
Social anxiety perceiving	0.00^*^
Distance learning is the cause of social anxiety	0.00^*^
Poor internet coverage	0.00^*^
Lacking suitable devices	0.01^*^
Year of study	0.04^*^
Faculty	0.44
Studying hours	0.08
Level of satisfaction	0.00^*^
Irregularity in lecture times	0.00^*^
Attended lectures while driving	0.00^*^
Level of grade	0.00^*^
Distance learning is boring	0.00*
Distance learning is time saving	0.00*
Distance learning has flexibility with time	0.00*
Better instruction	0.00*
Better interaction with instructors	0.00^*^
Better interaction with classmates	0.00^*^
Limitation in internet data	0.00^*^
Variation of educational platforms	0.06
GPA	0.45
Smartphone use	0.36
Hours spent in attending lectures	0.13
Quitting courses	0.00^*^
Attending lectures while not paying attention to it	0.00^*^
Level of study	0.00^*^
Lack the communicating with colleagues during distance learning	0.00^*^

**Table 6:** Tukey post-hoc analysis for determinants of total DELES score *P-value<0.05 DELES, Distance Education Learning Environments Survey

Predictor	I	J	P-value	Mean difference (I-J)	
Satisfaction level	Very dissatisfied	Dissatisfied	0.08	-6.23	
		Neutral	0.00^*^	-16.389	
		Satisfied	0.00^*^	-26.59	
		Very satisfied	0.00^*^	-53.15	
	Dissatisfied	Neutral	0.00^*^	-10.16	
		Satisfied	0.00^*^	-20.36	
		Very satisfied	0.00^*^	-46.92	
	Neutral	Satisfied	0.04^*^	-10.20	
		Very satisfied	0.00^*^	-36.77	
	Satisfied	Very satisfied	0.00^*^	-26.57	
Courses quitting	Never	Rarely	0.73	3.88	
		Sometimes	0.00^*^	11.38	
		Often	0.02^*^	11.69^*^	
		Always	0.00^*^	15.03	
					Rarely	Sometimes	0.18	7.50	
	Often	0.35	7.81		
	Always	0.11	11.14		
				Sometimes		Often	1.00	0.31	
						Always	0.92	3.64	
	Often	Always	0.96		3.33				
				Study hours		Less than 1 hour	1-2 hours	0.39	-1.43	
							3-4 hours	0.34	-1.44	
	More than 4 hours	0.00^*^	-3.34			
	1-2 hours	3-4 hours	1.00		-0.01	
		More than 4 hours	0.01^*^		-1.91	
	3-4 hours	More than 4 hours	0.00^*^		-1.90	
Comparison of grade level	Worse	Same	0.00^*^	-11.24	
		Better	0.00^*^	-13.190	
	Same	Better	0.00^*^	11.24	
Starting time of the lecture	Never	Sometimes	0.01^*^	7.71	
		Frequently	0.00^*^	13.36^*^	
	Sometimes	Frequently	0.11	5.65	
Attending lecture while not paying attention to it	Never	Sometimes	0.06	11.23	
		Frequently	0.00^*^	25.14	
	Sometimes	Frequently	0.00^*^	13.91	
Attending lecture while driving	Never	Sometimes	0.01^*^	7.50	
		Frequently	0.00^*^	21.42	
	Sometimes	Frequently	0.01^*^	-7.50	
Comparison of studying level	Worse	Same	0.00^*^	-13.00	
		Better	0.00^*^	-19.77	
	Same	Better	0.21	-6.77	
Communication with colleagues	Strongly disagree	Disagree	0.01^*^	20.12	
		Neutral	0.01^*^	18.64^*^	
		Agree	0.01^*^	18.76^*^	
		Strongly agree	0.00^*^	31.14^*^	
	Disagree	Neutral	1.00	-1.49	
		Agree	1.00	-1.37	
		Strongly agree	0.02^*^	11.01	
	Neutral	Agree	1.00	0.12	
		Strongly agree	0.00^*^	12.50	
	Agree	Strongly agree	0.39	1.01	

**Table 7:** Pearson's correlation coefficient analysis for determinants of DELES scores *P-value<0.05 DELES, Distance Education Learning Environments Survey

Variable	P-value	r
Age	0.00*	0.16
Number of courses	0.61	0.02

## Discussion

The aim of this study was to investigate distance learning quality during the COVID-19 pandemic. The study showed that 65.8% of the students were very dissatisfied or dissatisfied with the quality of distance learning, and the mean total DELES score was 61.6 ± 24.6. The highest mean of DELES score components was the instructor support component, while the lowest mean was for the active learning component. The results revealed that distance learning quality was determined by several factors such as student burnout and social anxiety, poor internet coverage, limited data availability, lack of suitable devices, negative attitudes of students toward distance learning, lack of communication with colleagues, irregular time of lectures, and distance learning satisfaction.

Our study showed that students who experienced social anxiety, lower communication with colleagues, and burnout had lower DELES scores compared to their counterparts. Previous studies showed that lockdown measures and reduced exposure to social situations were significantly associated with increase and maintenance of social anxiety symptoms among youth [13,14]. More than 60% of the participants agreed or strongly agreed that they barely communicate with their colleagues and self-perceived themselves as socially anxious during distance learning. The human factor is an essential component in any distance education environment, and the interaction between participants in the distance education environment is crucial to the development of a high-functioning distance education [15,16]. Consequently, both social anxiety and low communication with colleagues due to the pandemic restrictions could reduce the interaction between students during distance learning, thus resulting in lower distance learning quality. Moreover, burnout and low distance learning quality relationship could be considered bidirectional as previous studies showed that digital learning was associated with an increase of cynicism and emotional exhaustion and that both of them are considered dimensions of burnout [17]. In addition, low quality of distance learning will put students under a huge study burden that requires more effort to achieve the same level of education they had before distance learning, thus precipitating burnout among them. Furthermore, more than 80% of the students reported that they stopped attending their lectures and 20% of them had always or often encountered thoughts about quitting courses. Both of the previously mentioned variables were significantly associated with low quality of distance learning. These two behaviors may hugely affect the outcomes of the educational systems and result in incompetent graduates in different fields. In addition, students who more frequently reported that they drive while attending lectures had significantly lower DELES scores.

Studies showed that the implementation of distance learning faced many challenges, especially those arising from weak infrastructural, technological, institutional, and student elements such as poor internet quality, lack of infrastructure, and internet access [18]. Similarly, students in our study had reported many of the same obstacles such as poor internet coverage, limited devices, and data availability, and all of these factors were significantly associated with lower quality of distance learning. The negative attitudes of students toward distance learning, such as it being boring, time wasting, time rigid, and worse interaction with instructors and colleagues, significantly and negatively impacted the quality of distance learning reported by them using the DELES tool. Such negative attitudes were highly correlated in the literature with poor students' experience in learning [19]. Moreover, older age and year of study were positively associated with higher DELES scores. This suggests that students who have more experience in college education and in their field of study were less affected by the integration of distance learning amid the COVID-19 pandemic. In our study, students who reported low degree of satisfaction also reported lower quality of distance learning. In line with these findings, the literature showed that learning environment characteristics correlate with student satisfaction [12]. Furthermore, our study showed that students who had decreased performance and academic levels compared to the period before distance learning reported lower quality of distance learning. Likewise, studies showed that distance learning was associated with lower knowledge and academic performance than conventional learning [20,21]. Additionally, students who reported that they had irregular lecture times had significantly lower distance learning quality. Irregular lecture times was associated with unsuitability of time, and previous studies showed that irregular times of studying was associated with lower academic success [22].

In agreement with our results, previous studies conducted in different countries showed that students were dissatisfied with the quality and the quantity of distance learning [23,24]. During the early period of distance learning, students and teachers showed huge resistance and negative attitudes toward distance learning due to its unfamiliarity [25]. Learning theories showed that students' distance learning experience was correlated to their intentions and attitudes to use distance learning [19]. As a result, studies predicted that the quality of distance learning will improve when this resistance gradually declines and more governmental efforts are put into improving the infrastructure for distance learning [25]. Nevertheless, our study, which was conducted after a year and a half from the start of distance learning, showed that the quality of distance learning was still low.

Since the COVID-19 pandemic forced a shift from face-to-face teaching to distance learning, and several studies showed that there was a low satisfactory level of distance learning, more strategies must be implemented in order to become an effective medium for education [26]. First, incorporating advanced network technology and teaching methods to improve distance learning must be integrated, which was considered as an effective method to improve its quality [27]. Second, the use of hybrid methods of distance learning that combine both synchronous and asynchronous modes of distance learning as these modes complement each other and would help students to learn more from both of them [28]. Furthermore, student interaction and collaboration were considered as the predominant factor of positive outcome environment association [29]. However, a well-recognized limitation of distance learning was students’ isolation [29], and this was one of our major findings as the student interaction and collaboration component was one of the lowest scores from all DELES components. Thus, it was crucial to practice methods in distance learning that focus on student interaction and collaboration. Finally, it was important to use e-learning reliable models such as ADDIE model, ASSURE model, Merrill’s model, and Kemp model when designing and developing distance learning curriculum.

The cross-sectional design of this study limits inferences about causality and temporality between the distance learning quality and the determinants. The sample was taken only from a single institution in Jordan; thus, further studies with a multicenter design are recommended for future research. Another important limitation is the possibility of hidden confounding variables not addressed by the study. Further studies are recommended to consider more potential confounders in the relationship between distance learning and students’ characteristics. Although the use of a reliable tool for assessing the quality of distance learning (DELES) is considered a major strength in our study, the lack of cutoff points to classify the quality of distance learning is a limitation as it limited our ability to perform binary or multinomial regression analysis. In addition, we were not able to perform linear regression analysis due to the high risk of multicollinearity between the independent variables and the lack of linearity between the dependent and independent variables. Lastly, the use of self-administered questionnaires is considered a limitation as such tools carry the risk of recall bias.

## Conclusions

To summarize, the emergence of the COVID-19 pandemic led to the incorporation of distance learning instead of face-to-face teaching resulting in major changes in the quality of learning. Our study showed that the quality of distance learning was low, and most of the students reported high levels of dissatisfaction. Furthermore, the study showed that distance learning quality was determined by several factors related to general demographics, and students’ perceptions and attitudes. In consideration of the aforementioned evidence, our study recommends the improvement of distance learning infrastructures and the implementation of adjunct learning methods that can enhance the quality of distance learning.
